# Knowledge and compliance related to the prevention of mother-to-child transmission guidelines amongst South African healthcare professionals

**DOI:** 10.4102/phcfm.v18i1.5304

**Published:** 2026-05-19

**Authors:** Ayabonga Vika, Burt Davis

**Affiliations:** 1 Africa Centre for Inclusive Health Management, Faculty of Economic and Management Sciences, Stellenbosch University, Stellenbosch, South Africa

**Keywords:** vertical transmission prevention guidelines, prevention of mother-to-child transmission, South Africa, knowledge, compliance, implementation, barriers

## Abstract

**Background:**

Prevention of Mother-to-Child Transmission (PMTCT) guidelines provide evidence-based protocols to prevent infections such as human immunodeficiency virus (HIV) from passing from mother-to-child. Regular updates ensure alignment with evolving treatments and best practices. It is therefore essential that all healthcare professionals clearly understand and consistently follow the latest PMTCT guidelines. So far, there seems to be limited research that explored healthcare professionals’ knowledge and practices regarding the PMTCT guidelines in South Africa.

**Aim:**

This study aimed to assess healthcare professionals’ knowledge and compliance related to the South African 2023 PMTCT guidelines.

**Setting:**

Healthcare professionals involved in antenatal and postnatal care across public and private healthcare facilities in all South Africa’s provinces.

**Methods:**

A cross-sectional study of 221 participants (35 doctors, 77 nurses and 109 clinical associates) was conducted over 8 weeks using an online survey distributed via social media.

**Results:**

Participants generally demonstrated high knowledge of the PMTCT guidelines, with significantly higher scores among those who had received formal training. Some knowledge deficits, e.g. in HIV testing procedures and infant antiretroviral dosing, remain. Doctors showed significantly higher knowledge scores than nurses and clinical associates. Fewer than half of the participants reported consistent compliance.

**Conclusion:**

While knowledge of the PMTCT framework was high, low compliance remains.

**Contribution:**

This study provides foundational information on the knowledge of healthcare professionals across various South African provinces about the 2023 PMTCT guidelines, highlights the vital role of clinical associates and stresses the need for consistent and structured PMTCT training.

## Introduction

Prevention of mother-to-child transmission (PMTCT) programmes have become crucial in halting the generational spread of human immunodeficiency virus (HIV), with as much as 90% of all HIV infection in children being through vertical transmission.^1^ In a country such as Cuba, PMTCT programmes have shown great success. Cuba eliminated vertical transmission of HIV a decade ago, receiving World Health Organization (WHO) validation in 2015.^2^ In Africa, Botswana was validated by the WHO in 2021 as the first high HIV-burden country, globally and in Africa, to eliminate vertical transmission of HIV.^3^ The efficacy of PMTCT programmes is exemplified by countries such as Cuba and Botswana. With sustained efforts, South Africa is also well-positioned to reach the PMTCT milestones these countries have achieved.

In South Africa, continued improvements in PMTCT programmes have resulted in a decline in vertical transmission rates from 23% in 2003 to less than 1% by 2019.^4^ However, the story is not one of linear success. South Africa had a 3.9% vertical transmission rate in 2021, and the paediatric HIV incidence rate was above 750 per 100 000 births.^5^ By 2023, the vertical transmission rate had slightly improved to 3.5%, signalling that gains are fragile and continued vigilance is required.^6^ One way to help safeguard continuous improvement is by making sure that healthcare professionals clearly understand and consistently follow the latest PMTCT guidelines.

Prevention of mother-to-child transmission guidelines are important because they provide evidence-based strategies and protocols to reduce the risk of transmitting infections, such as HIV, from mother-to-child during pregnancy, labour, delivery and breastfeeding.^7^ Such guidelines help healthcare professionals to implement effective programmes, improve maternal and infant health outcomes and align practices with international standards, ultimately reducing the incidence of vertical transmission of communicable infections.^7^ If healthcare professionals are not up-to-date on the newest PMTCT guidelines, the consequences can be serious: missed opportunities for early diagnosis, inappropriate treatment, and ultimately preventable HIV infections in new-borns.^8^

This begs the question: how knowledgeable are healthcare professionals in South Africa on the PMTCT guidelines? Moreover, to what extent are they complying with these guidelines? In this study, we explore these potential concerns associated with South Africa’s PMTCT guidelines by investigating the knowledge and compliance of local healthcare professionals in this regard.

Prevention of mother-to-child transmission guidelines in South Africa continue to evolve in line with new treatments, diagnostics and global best practice.^9^ The national PMTCT programme began in 2002, with guidelines amended in 2004 and 2013.^10^ The most recent major update came in 2023, when the guidelines – renamed the Guideline for Vertical Transmission Prevention (VTP) of Communicable Infections – introduced an integrated approach to preventing HIV, syphilis, hepatitis, malaria and tuberculosis.^9^ Key components include universal early HIV testing, immediate Antiretroviral Therapy (ART) initiation and a dolutegravir-based (TLD) regimen. The guidelines also emphasise strengthened antenatal and postnatal care, family-centred services and partner involvement.^9,11^

So far, there seems to be limited research which have explored healthcare professionals’ knowledge and practices related to the PMTCT guidelines in South Africa. Earlier studies from Ogbonna and Mogano at Odi Hospital in Tshwane District showed moderate healthcare worker knowledge of PMTCT guidelines.^12,13^ Ogbonna reported an average knowledge score of 60.8%, with good procedural understanding (average 77% practice compliance) but weak medication-related knowledge, especially regarding ART combinations and dosing. Mogano found a similar pattern, with participants’ knowledge score at 69% overall, showing reasonable awareness of HIV transmission and counselling but limited knowledge of recent guideline updates. Notably, formal training did not significantly improve knowledge in that study.

A later South African national survey by Chisholm, involving nearly 2000 healthcare workers across professions, highlighted ongoing pharmacological knowledge gaps, particularly around dolutegravir drug interactions.^14^ Although not focused specifically on PMTCT, it underscored broader challenges in translating updated HIV guidance into practice. The most recent work by Magugu in two KwaZulu-Natal referral hospitals reported much higher knowledge scores – median 91.7% among 249 doctors and nurses – despite fewer than half having formal training.^15^ However, gaps remained in HIV testing frequency and antenatal screening, prompting calls for structured, inter-professional training to reinforce guideline compliance.

Taken together, these studies suggest that while healthcare professionals generally have foundational PMTCT knowledge, up-to-date and detailed understanding often lags frequent guideline revisions. Given the ever-evolving nature of PMTCT guidelines especially in South Africa, up-to-date data on healthcare professionals and how they engage with these guidelines remains crucial for sustaining national progress in preventing mother-to-child transmission.

Given this scenario, the aim of this study was to identify knowledge-related, compliance related and implementation gaps associated with South Africa’s PMTCT guidelines, by gauging the perspectives of healthcare professionals in the country.

## Research methods and design

### Study design and sampling

A cross-sectional study was conducted to assess healthcare professionals’ knowledge of South Africa’s 2023 PMTCT guidelines. Data were collected via a self-administered online survey consisting of close-ended questions.

Purposive sampling was employed given that participants had to be knowledgeable about or have direct experience with the PMTCT guidelines. The study specifically targeted clinical healthcare professionals – nurses of all cadres, medical doctors and clinical associates working in antenatal and postnatal care across both public and private healthcare facilities in South Africa. (There is an estimated population of 245 117 healthcare professionals in the country.)^16,17,18^ Additional inclusion criteria required participants to be ≥ 18 years, practicing in South Africa, and providing informed consent. Allied health professionals and non-South African healthcare professionals were excluded. Recruitment occurred through professional and social media networks (WhatsApp, LinkedIn, Facebook and Instagram), with reminders posted bi-weekly over 8 weeks.

We aimed fora a moderate effect size of 0.5 with an α of 0.05 (two-tailed) and a β of 0.20 and wanted to determine the effects of various independent variables (with two or three groups each, see Table 1) on the dependent variable *knowledge score*, respectively, using *t*-tests and analysis of variance (ANOVAs). This meant that we required a minimum of 130 participants (in cases of independent variables with two groups) and a minimum of 160 participants (in cases of independent variables with three groups) to ensure reliable, statistically significant results detecting meaningful differences in our studied population.^19,20^ We therefore targeted a total sample size of at least 160 participants.

**TABLE 1:** Demographic characteristics of the participants (*N* = 221).

Variable	Category	*n*	%
Formal training on PMTCT guidelines	Yes	96	43.4
	No	125	56.6
Designation	Clinical associate	109	49.3
	Nurse	77	34.8
	Doctor	35	15.8
Gender	Female	166	75.1
	Male	52	23.5
	Prefer not to say	3	1.4
Age group (years)	< 30	145	65.6
	≥ 30	76	34.4
Years of practice	≤ 1–5	144	65.2
	6–10+	77	34.9
Sector of employment	Public	86	38.9
	Private	120	54.3
	Prefer not to say	15	6.8
Province	Gauteng	123	55.7
	Eastern Cape	29	13.1
	Free State	5	2.3
	KwaZulu-Natal	15	6.8
	Limpopo	4	1.8
	Mpumalanga	7	3.2
	North West	12	5.4
	Northern Cape	3	1.4
	Western Cape	6	2.7
	Prefer not to say	17	7.7

PMTCT, prevention of mother-to-child transmission.

### Data collection tool

The researcher-developed survey items were based on the Guideline for VTP of Communicable Infections and ART Clinical Guidelines. Validity was ensured through expert review and pilot testing. A medical doctor from a perinatal HIV research unit reviewed the tool to confirm that it appropriately assessed knowledge aligned with PMTCT guideline themes and supported the research aims. The doctor evaluated each question’s relevance using a 4-point scale (1 = *not relevant*; 2 = *somewhat relevant*; 3 = *quite relevant*; and 4 = *highly relevant*). All questions were either rated as 3 or 4 and considered ‘relevant’. A pilot study with nine healthcare professionals (three doctors, three nurses and three clinical associates) provided feedback on question style and demographic items, which were revised accordingly. The survey length was positively received as short, and participants were satisfied with the clarity of the questions.

Reliability was strengthened through expert review and pilot testing by clarifying unclear items, adjusting the difficulty of knowledge questions, standardising instructions, ensuring consistent scoring and including sufficient items to measure each PMTCT-related variable or theme. We used inter-rater reliability to measure the degree to which the expert reviewer and nine healthcare professionals gave consistent answers when tasked with indicating whether the various items measured each PMTCT-related theme or not. Using simple agreement ratio (% agreement), for all items related to the various themes, respectively, there were at least ≥ 80% level of agreement between the observers.

### Measures

#### Demographic information

Participants were asked to indicate whether they received formal training on PMTCT guidelines, their designation, gender, age group, years in clinical practice, sector of employment, and the province they reside in (See Table 1).

#### Knowledge score

Knowledge was measured with an 18-item multiple choice questions or items focusing on the PMTCT guidelines. It covered the following themes: HIV testing, pre-exposure prophylaxis (PrEP), post-exposure prophylaxis (PEP) and antiretroviral regimens. Each correct answer was scored as 1, while incorrect answers and no answers provided were scored as zero. Responses were scored by tallying correct answers.

#### Prevention of mother-to-child transmission guidelines compliance level

Participants completed a researcher-designed question about their perceived level of compliance with the PMTCT guidelines (1 = *Always*, 2 = *Often*, 3 = *Sometimes*, 4 = *Rarely*, 5 = *Never*).

### Data analyses

Data were exported and analysed using Excel, SPSS 30 and RStudio. For the closed-ended questions, descriptive statistics (means, standard deviations, frequencies and percentages) summarised knowledge scores and demographics. As mentioned, inferential analyses included *t* tests and ANOVAs to examine associations between *knowledge score* and demographic variables *formal training on PMTCT guidelines, designation, gender, age group, years in clinical practice* and *sector of employment*, respectively (5% margin of error; a 95% confidence level).

### Ethical considerations

Ethical clearance was obtained from Stellenbosch University’s Research Ethics Committee: Social, Behavioural and Education Research (REC: SBE) (project number: 30931) in South Africa. Participants provided online consent before completing the survey as part of the same process on a secure university approved platform, Stellenbosch University (SUN) surveys. Online consent consisted of ticking a box prior to starting the survey. To ensure confidentiality, access to the survey was limited to the investigator and was secure and password protected on the same platform. Anonymity was ensured by using anonymous SUN survey links and by excluding the collection of any identifying information, e.g. personal identifiers such as names, contact details or internet protocol (IP) addresses. As a result, individual responses could not be linked to participants, which meant that incomplete surveys could not be followed up and reminder e-mails could not be sent to those who did not respond. Lastly, data were reported on in an aggregate form.

## Results

### Demographic information

A total of 221 healthcare professionals participated in the study, with the majority being clinical associates (49.3%; *n* = 109). For designation, we initially asked nursing participants to indicate whether they were a registered nurse, an enrolled nurse or an enrolled nurse assistant, but because of unequal representation across nursing ranks (only one enrolled nursing assistant took part), we decided to combine these participants into one group, ‘nurse’.

The sample was predominantly female (75.1%; *n* = 166) and young, with 65.6% of participants under the age of 30 years (*n* = 145). Most respondents had less than 5 years of practice experience (65.2%; *n* = 144). More than half of the participants (56.6%; *n* = 125) reported not having received formal training on the PMTCT guidelines, highlighting a potential training gap among frontline providers. In terms of sector of employment, most participants worked in the private sector (54.3%; *n* = 120). There were participants from all provinces, most prominently from Gauteng province (*n* = 123) (see Table 1).

#### Knowledge score

**FIGURE 1:**
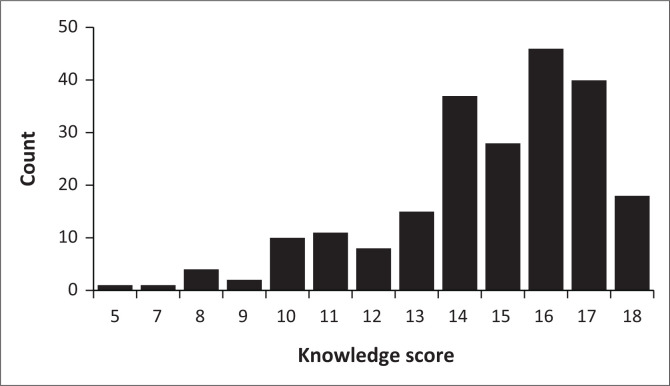
Bar chart of knowledge score distribution.

The overall score for knowledge was 14.71. (All variables were measured on an 18-item scale.) See Figure 1 for a distribution of the scores.

The difficulty or ease index – a quantitative index applied in educational measurement to evaluate the relative level of difficulty of a specific test item – suggests that the percentage of participants in multiple choice questions who correctly answers an item should range from 30% to 70%.^21^ Given the relatively high knowledge score, it is not surprising that most of the items fell well beyond this range (70%+), except in four cases – Items 7, 12, 13 and 16 – where less than 70% participants gave the correct answer. See Table 2 for details of these four items.

**TABLE 2:** Prevention of mother-to-child transmission knowledge items 7, 12, 13 and 16.

Item	Knowledge item	Correct answer	Correct responses (%)	Incorrect responses (%)
7	When should HIV testing be performed on pregnant women?	At first antenatal visit, with repeat testing during pregnancy and breastfeeding	63.35	36.65
12	What is the most appropriate HIV confirmation test for a child aged 18 months to 2 years?	HIV antibody test (rapid test or ELISA)	56.11	43.89
13	In infants known to be HIV exposed, an HIV infant test is required at 6 days (True/False).	False	69.68	30.32
16	How many times a day should a high-risk HIV exposed infant receive Zidovudine?	Twice daily	53.39	46.61

HIV, human immunodeficiency virus.

**FIGURE 2:**
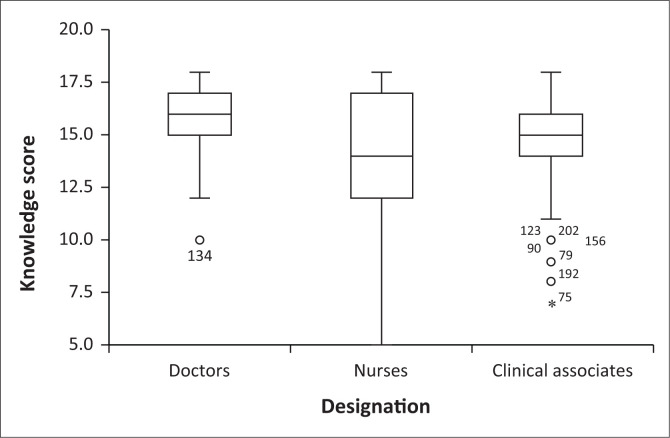
Box and whisker plot comparing knowledge score distribution across designation. The black centre line denotes the median value (50th percentile), with the white box (25th to 75th percentiles), and the black whiskers (5th and 95th percentiles). The values beyond the upper and lower bounds are considered outliers.

We then conducted an ANOVA to test the effect of the independent variable *designation* on the dependent variable *knowledge score*. A significant effect of *designation* on *knowledge score* was found F(2,218) = 6.54 (*p* < 0.01; η^2^ = 0.057). Post hoc tests showed a significant difference (*p* < 0.01) between doctors (*M* = 15.94; standard deviation [*s.d*.] = 1.81) and clinical associates (*M* = 14.72; *s.d*. = 2.18) and between doctors (*M* = 15.94; *s.d*. = 2.18) and nurses (*M* = 14.14; *s.d*. = 2.98). There was no significant difference (*p* > 0.01) between clinical associates and nurses. See Figure 2 for a visual representation of the data.

**FIGURE 3:**
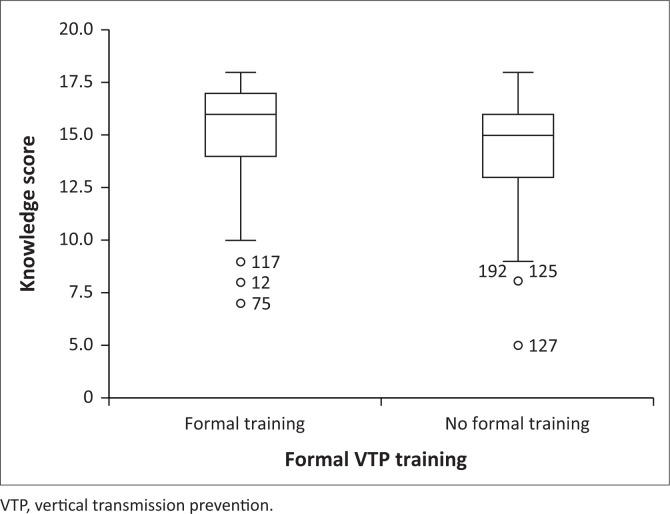
Box and whisker plot comparing knowledge score distribution across formal training on the prevention of mother-to-child transmission guidelines. The black centre line denotes the median value (50th percentile), with the white box (25th to 75th percentiles) and the black whiskers (5th and 95th percentiles). The values beyond the upper and lower bounds are considered outliers.

Next, a *t*-test was conducted to find possible differences in *knowledge score* between participants who had received formal training on the PMTCT guidelines and those who had not. A significant effect of *formal VTP training* on *knowledge score* was found *t*(219) = 2.83 (*p* < 0.01; η^2^ = 0.384). Participants who had received formal training had a higher knowledge score (*M* = 15.25, *s.d*. = 2.38) compared to participants who had not received formal training (*M* = 14.30, *s.d*. = 2.52). See Figure 3 for a visual representation of the data.

Lastly, *t*-tests were conducted to test the effects of the independent variables *gender, age group, years in clinical practice* and *sector of employment* on the dependent variable *knowledge score*, respectively. No significant results were found in any of the cases.

#### Prevention of mother-to-child transmission guidelines compliance level

Participants’ self-reported responses revealed variability in the frequency of compliance with the PMTCT guidelines in clinical practice. While a minority reported consistent adherence (*n* = 87; 39%), a substantial proportion indicated partial or infrequent compliance (*n* = 134; 61%). See Table 3 for a detailed breakdown.

**TABLE 3:** Frequency of self-reported compliance with prevention of mother-to-child transmission guidelines.

Frequency of compliance	*n*	%
Always	87	39
Often	39	18
Sometimes	35	16
Rarely	38	17
Never	22	10

## Discussion

This study set out to assess healthcare professionals’ knowledge and compliance with the implementation of the PMTCT guidelines. To appreciate the study’s main findings, it is important to understand the demographic profile of the participants enrolled in this study. Overall, the demographic profile of the participant sample suggested a young, predominantly female, and relatively inexperienced cadre of healthcare professionals, the majority of whom was clinical associates lacking formal training in PMTCT guidelines who mostly worked in the private sector and hailed from Gauteng province.

Main findings showed generally high knowledge scores related to the PMTCT guidelines among all cadres. A total of 14 out of 18 items were correctly answered by more than 70% of participants which, according to the difficulty index, indicates that questions might have been too easy.^21^ However, given that a lack of high knowledge levels about some of the issues could have dire health consequences for patients and that the questionnaire was validated through a pilot study and expert review, the high knowledge scores were reassuring. Moreover, knowledge of these guidelines is imperative for realising Sustainable Development Goal 3, which ensures healthy lives and promotes well-being for all. Knowledgeable healthcare professionals will in turn be instrumental in decreasing the avertable transmission of HIV from mother-to-child. Perhaps to be expected given their higher level of training, medical doctors had significantly higher knowledge scores compared to nurses and clinical associates. No differences were found for knowledge scores based on participants’ gender, age group, years of experience or sector of employment.

Notably, the study found that formal training on the PMTCT guidelines plays a vital role, as those participants who received formal training scored significantly higher for knowledge on the guidelines compared to those who did not. This finding may be indicative of why a major theme for improving the implementation of the PMTCT guidelines identified by participants was the need for regular and mandatory training on these guidelines. Additionally, this finding calls on policymakers to consider compulsory training for all health cadres involved in the clinical management of patients.

Alarmingly, fewer than half of the participants indicated that they consistently comply with the PMTCT guidelines – a notable concern given the critical role it plays in preventing mother-to-child HIV transmission. Non-compliance with the guidelines has dire implications such as harm to patient health, missed opportunities for patient care as well as the potential for drug resistance.

The findings of this study align with and extend the existing body of work related to knowledge of PMTCT guidelines discussed earlier. The overall score for knowledge was 14.71 out of 18. Put differently, it means that the average score among participants was 81.72%, indicating substantial improvement compared with earlier PMTCT-focused research conducted by, for example, Ogbonna in 2016.^12^ As with Ogbonna, knowledge of drug dosages and combination therapy remained an area of weakness, with nearly half of participants answering the Zidovudine dosing question incorrectly.^12^ In contrast to Mogano’s finding that prior training had no significant impact on knowledge, this study revealed a statistically significant association between formal training and higher knowledge scores, suggesting that training initiatives under the updated PMTCT framework are effective in enhancing guideline comprehension and application.^13^

### Strengths of the study

Firstly and most importantly the strength of this study lies in the scope and inclusivity of this research. Unlike prior studies, which were mostly limited to single hospitals, provinces or professions, this study included doctors, nurses and clinical associates across multiple provinces, offering foundational information on the knowledge healthcare professionals across various South African provinces have of the 2023 PMTCT guidelines. By doing so, it fills an important gap in the literature and provides a comprehensive overview of healthcare professionals preparedness to implement the most recent 2023 PMTCT guidelines. Secondly, clinical associates formed the largest cohort of study participants in the data set and performed well in the survey. Clinical associates are a new cadre in the South African healthcare setting.^18^ Our findings clearly indicate that this group can have a positive impact on the South African health landscape and should be tapped into for future HIV or primary healthcare programmes. Thirdly, this study – at the time of writing – provides the most up-to-date data on the knowledge of healthcare professionals in South Africa on the latest PMTCT guidelines, serving as a baseline for future studies in this regard.

### Limitations of the study

This study was not without its limitations. Firstly, a survey consisting of novel items was used to assess healthcare professionals’ knowledge of the 2023 PMTCT guidelines, which limited comparability with data collection tools from similar studies and made it difficult to establish construct validity. However, the tool was developed using items derived from the national Department of Health (DoH) guidelines and underwent expert review and pilot testing. Secondly, participants reported on their perceived level of compliance with the PMTCT guidelines, which may have introduced a potential level of bias. It was unfortunately not within the ambit or budget of the study to measure actual compliance. Sampling bias may also have occurred given that the majority of the participants were relatively junior. Nevertheless, considering the high knowledge scores for the group, it was encouraging that younger, less experienced healthcare professionals displayed good knowledge of the PMTCT guidelines. Sampling bias because of the electronic survey used may also be possible, potentially excluding healthcare professionals who do not have access to the internet. Thirdly, 597 individuals accessed the survey, but only 221 (37%) completed it. As discussed earlier, because of its voluntary and anonymous nature, reminder follow-ups were not possible. Lastly, also as discussed before, unequal representation across nursing ranks constrained subgroup analyses. Nursing categories were therefore combined to maintain statistical validity.

### Implications for policy and practice

The findings of this study carry several practical and policy-level implications for strengthening VTP efforts in South Africa. Firstly, the demonstrated association between formal training and higher knowledge scores underscores the importance of regular, structured and competency-based training programmes for all healthcare cadres involved in maternal and child health services. Integrating such training into ongoing professional development could ensure more consistent comprehension of and compliance with the evolving PMTCT guidelines. Secondly, the recurring knowledge deficits in HIV testing procedures and infant antiretroviral dosing identified in this study highlight the need for targeted refresher training and supervision mechanisms that address these specific areas. Strengthening supportive supervision and mentorship in clinical settings may further enhance translation of theoretical knowledge into practical competence. Thirdly, the inclusion of diverse professional groups – including nurses, doctors and clinical associates – in PMTCT implementation and monitoring aligns with the principles of inter-professional collaboration and integrated care promoted in national policy frameworks.^22^ Finally, to sustain high levels of knowledge and guideline compliance, it is essential that institutional and provincial health authorities facilitate timely dissemination of guideline updates, ensure equitable access to reference materials and integrate PMTCT performance indicators into routine quality improvement systems. Collectively, these measures could enhance the fidelity of PMTCT guideline implementation, promote continuity of care across service levels and accelerate national progress towards the elimination of vertical transmission of HIV and related infections.

## Conclusion

The first key takeaway from this study suggests that healthcare professionals were well-versed on the PMTCT framework, but continued attention is needed to address persistent knowledge gaps in specific clinical area. The second key takeaway is that improving compliance with the PMTCT guidelines is urgently required. To do so, the study’s findings point to the solution requiring an integrated approach that combines capacity building, systemic support and practical implementation tools. Sustained progress in preventing vertical HIV transmission will depend not only on knowledge dissemination but also on the creation of a supportive clinical environment that empowers healthcare professionals to apply these guidelines confidently and consistently.
